# Advances and Challenges of Self-Healing Elastomers: A Mini Review

**DOI:** 10.3390/ma15175993

**Published:** 2022-08-30

**Authors:** Jun Xu, Lei Zhu, Yongjia Nie, Yuan Li, Shicheng Wei, Xu Chen, Wenpeng Zhao, Shouke Yan

**Affiliations:** 1 School of Chemical Engineering, Qingdao University of Science & Technology, Qingdao 266042, China; 2 Key Laboratory of Rubber-Plastics, Qingdao University of Science & Technology, Qingdao 266042, China

**Keywords:** fast self-healing, elastomers, healing mechanism

## Abstract

In the last few decades, self-healing polymeric materials have been widely investigated because they can heal the damages spontaneously and thereby prolong their service lifetime. Many ingenious synthetic procedures have been developed for fabricating self-healing polymers with high performance. This mini review provides an impressive summary of the self-healing polymers with fast self-healing speed, which exhibits an irreplaceable role in many intriguing applications, such as flexible electronics. After a brief introduction to the development of self-healing polymers, we divide the development of self-healing polymers into five stages through the perspective of their research priorities at different periods. Subsequently, we elaborated the underlying healing mechanism of polymers, including the self-healing origins, the influencing factors, and direct evidence of healing at nanoscopic level. Following this, recent advance in realizing the fast self-healing speed of polymers through physical and chemical approaches is extensively overviewed. In particular, the methodology for balancing the mechanical strength and healing ability in fast self-healing elastomers is summarized. We hope that it could afford useful information for research people in promoting the further technical development of new strategies and technologies to prepare the high performance self-healing elastomers for advanced applications.

## 1. Introduction

Polymeric materials have become one of the most widely used structural and functional materials due to their light weight, high stretchability, good processability, and multi-functionality [1]. However, compared with metals and ceramics, polymeric materials exhibit a poor mechanical strength, which makes them vulnerable to damage [2]. The creation of structural injuries will unambiguously lead to a significant decline in the mechanical properties and functions, or even to the failure of the related products for further service, which severely limited their practical applications [3,4]. Endowing the polymeric materials with a self-healing capability will certainly improve the stability and safety of them in practical applications and prolong their lifetime. Therefore, the design of self-healing polymeric materials with multiple functions has attracted increasing interest [5,6,7,8,9,10,11,12,13].

**Figure 1:**
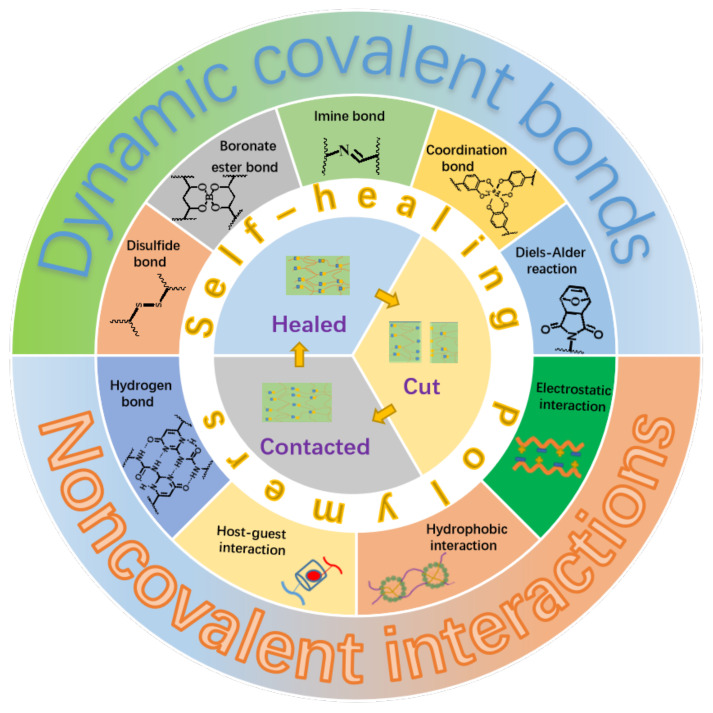
Illustration of self-healing elastomers based on different dynamic covalent bonds or/and non-covalent interactions.

Generally, the self-healing polymers can be divided into two categories depending on their self-healing natures. The first category is named as extrinsic system, which is fabricated by embedding microcapsules with reactive fluids or agents within the polymer matrix [14]. The self-healing is realized in a way that the reactive fluids or agents flow out upon injury of the microcapsules caused by damage, which subsequently trigger the in-situ chemical reactions to heal the damaged parts. It is clear that the self-healing ability will lose after completely exhausting of the reactive fluids or agents. For the other category, as illustrated in Figure 1, the materials themselves contain some kinds of reversible dynamic covalent bonds, such as the Diels–Alder reaction and disulfide bond [15,16], or/and various kinds of physical interactions, such as metal-ligand, hydrogen bond, and electrostatic interactions [17,18,19,20,21,22], which makes the self-healing to be an intrinsic nature of the materials. This kind of material is referred to as an intrinsic system and can heal the damages repeatedly. Moreover, the self-healing polymers can be categorized into autonomic and non-autonomic ones depending upon the initiating condition or pathway of the self-healing. It is not hard to understand literally that the autonomic one can repair damages spontaneously through chemical reactions or recovery of the intermolecular interactions without the need of any kind of external stimuli. The other one, the repair of damaged polymers can only be achieved through some kinds of external stimuli such as the heat and light.

Even though the concept of self-healing polymer was first proposed in 1950s [23], the development of it was insufficient until the 21st century. The polymeric materials with self-healing ability have then attracted much attention since 2000, and great progress has been achieved during the last 20 years. Its development can be divided into realization, blossoming, strength, mild-condition, and fast-healing stages, according to the chronological advancement, namely the perspective of their research priorities at different periods. The first stage focuses mainly on the realization of polymers with self-healing ability, while performances related to other aspects, e.g., tensile strength and functionality, are of less concern. Along with the appearance of self-healing polymer, the investigation of it has blossomed quickly [24]. In the stage referred here to as “strength”, attentions were paid on the mechanical properties of self-healing polymers in order to satisfy the demand of practical applications. At this time, a great number of self-healing polymers with high tensile strength have been reported [25,26,27,28]. While it is recognized that the self-healing of materials with high tensile strength takes place generally at high temperature, sometimes higher than 100 °C, which limited their application, the development of self-healing polymers moves to the fourth stage referred to as “mild-condition” in this paper. Among many ingenious designs such as mechano-responsive strategy [29], novel dual physical cross-linked network [30], molecular engineering of hard domains [31], and mechano-responsive hydrogen-bonding array [32], a milestone work in this stage achieved by Sun et al. [33] shows that the polymeric composite with a high tensile strength of 81 MPa can achieve 99% healing efficiency with the assistance of immersing in 45 °C water. Even ultra-robust (53 MPa) materials with high healing efficiency (80–100%) at room temperature has been reported by Zhang et al. [34].

It should be noted that with the rapid progress of bio-integrated electronics, such as, electronic skins, implantable electronics, and wearable sensors, the flexible electronics based on elastomers have drawn increasing interest [35,36,37,38]. However, the healing time of self-healing elastomers is usually consuming (sometimes lasting for few hours), which results in a long-term failure of the electronics [39]. Consequently, the development of self-healing elastomers with rapid healing ability is highly desired. This makes the design of fast self-healing elastomers, ideally with real-time healing ability at ambient environment, becomes a challenge in this field. In this review, after a brief introduction of the self-healing process for the polymers, the advances of self-healing elastomers with fast healing speed less than 2 h will be summarized in detail. The future development direction and remaining challenges of this field will be looked forward, finally.

## 2. Healing Process of Intrinsic Self-Healing Polymers

Due to the potential technological relevance in various fields of self-healing polymers and the substantial for sustainable development, endowing the polymers with self-healing capability and exploring the fundamental mechanism underlying the healing process have attracted increasing interest [40,41,42,43]. In early 1980s, a theory of crack healing of polymers was first established by Wool et al. [44,45] in which the crack healing process of thermoplastic polymers was divided into five parts, termed surface rearrangement, surface approach, wetting, diffusion, and randomization. In a fresh crack surface, the surface rearrangement is mainly in the forms of topographic evolution or roughness of the surface, molecular weight distribution, and chain-end distributions [46]. Plus, compared to the linear molecules, the star-shape ones can form more time-stable networks. [47] Suitable pressure is applied in “surface approach” process to ensure the two freshly damaged surfaces contacting closely together in order to form an interface and wetting each other prior to the molecular level diffusion. The molecular level diffusion plays a key role in the formation of new interactions at the interfaces, which regulates the healing strength of healed polymers. To understand the diffusion at interfaces, the reptation model was used to investigate the healing at polymer–polymer interfaces, which depicted the decrease of healing rates with increasing molecular weight in a power law dependency and the proportional dependence of healing strength on the average interpenetration distance [48,49]. It should be pointed out that the self-healing process of the intrinsic system based on various dynamic bonds or/and different kinds of physical interactions is much more complex due to the multiple and diverse dynamic interactions in different individual systems [50].

Taking the self-healing polymer based on hydrogen bond as an example, the supramolecular polymers with self-healing capability was first established by Cordier et al. [51], who designed a self-healing supramolecular rubber based on small molecules assembled through hydrogen bonds. The supramolecular rubber could recover completely its mechanical properties after 3 h healing at room temperature. It is well documented that the strength of the associations based on the hydrogen bonds is lower than the one on the covalent bonds. Therefore, a large number of dissociated hydrogen bonds present at the fracture surfaces as broken, which endow the supramolecular rubber with efficient self-healing ability through reforming of the hydrogen bonds. This proposed mechanism of self-healing has been subsequently verified by testing the healing efficiency of the samples after being healed for different waiting times. The fact that the healing efficiency decreases with increment of waiting time reflecting the reduction in number of dissociated groups owing to the formation of new bonds within the broken surface during waiting, and thus the reduction of non-associated groups on the fracture surfaces available for self-healing. The aging-time-dependent healing efficiency has, actually, been observed for the majority of the reported self-healing polymers based on hydrogen bond [52,53,54]. Moreover, apart from hydrogen bond based self-healing polymer, self-healing polymers assembled through metal-ligands or ionic interactions also exhibit the similar aging sensitivity [55,56,57]. It was ascribed to the water molecules in air which take part in a series of irreversible processes by coordination with metal ions or ion clusters destroying the noncovalent cross-linkers. It is clear that the evolution of dynamic bonds on the fracture surface plays an important role in surface rearrangement stage for the self-healing polymers based on dynamic bonds.

In the self-healing process, when the two fractured surfaces were brought into contact, the wetting and the reversible bonds re-association took place at the interfaces prior to the molecule-level diffusion [46]. Interestingly, in the systems without dynamic bonds exchange, wetting can also impact the subsequent self-healing stage, and even determine the kinetics of healing process [58]. In order to obtain good wetting, almost all of the self-healing polymers based on hydrogen bonds were applied a certain pressure on the damaged interfaces during the self-healing process. [29,30,31,32]. Moreover, except for the physical interactions based self-healing polymers, dynamic covalent bonds, e.g., disulfide bond and boron-based bond, based self-healing polymers also needs external pressure during the self-healing process to achieve satisfactory self-healing performance (see Table 1) [59,60,61,62,63,64]. A recent study based on experimental and theoretical simulations suggests that the compressive force on the healing interfaces maybe attenuate the activation energy barrier of dynamic bond exchange that can further promote the healing in the manner of accelerating dynamic exchange [65].

**Figure 2:**
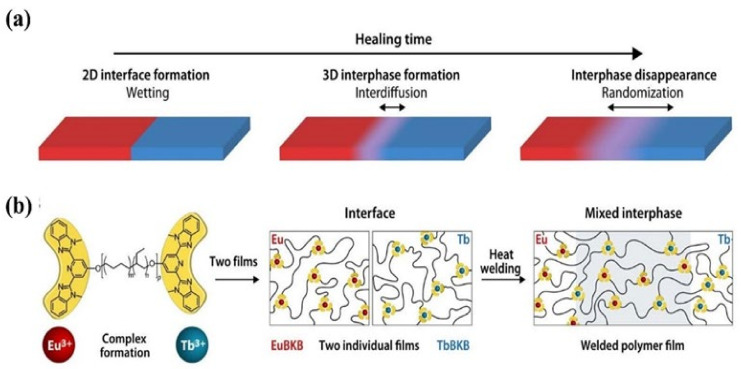
The healing process in polymers. (**a**) The final stages of the healing process in polymers involve wetting, interdiffusion with re-entanglement, and randomization. (**b**) To investigate the healing process on a length scale of a few nanometers, metallosupramolecular polymers (MSPs) assembled from telechelic PEB with terminal Mebip ligands (*Mn* = 3800 g mol^−1^; *m* ≈ 0.32, *n* ≈ 0.68, *p* ≈ 55) and either Eu(ClO_4_)_3_ or Tb(ClO_4_)_3_ were studied. The two metallosupramolecular polymers display similar properties, but the different ion types can be monitored in a spatially resolved manner. Reproduced with permission [69]. Copyright 2021, American Association for the Advancement of Science.

We recall that the molecular-level diffusion is essential for self-healing, and the damaged sample can only recover its initial mechanical properties when the healed interfaces exhibit indistinguishable structure from the pristine sample [66]. Quantitative study on the evolution of the healed interfaces, especially the interfacial diffusion in microscopic scale during the healing process is, however, difficult. Therefore, the macroscopic experiments, e.g., disappearance of scratches, restoration ratio of tensile strength and strain are used to evaluate the healing performance of self-healing polymers. Moreover, the internal reflection infrared imaging [67] and laser speckle imaging [68] have been used to explore the microscopic healing process, even though their resolutions are not high enough. Recently, Schrettl et al. [69] reported a unique stratagem to monitor the interfacial diffusion in healing process with a high resolution of a few nanometers by energy-dispersive X-ray (EDX) spectrum imaging using scanning transmission electron microscopy. Heterogeneous interfaces as illustrated in Figure 2a were constructed to investigate the interfacial diffusion of the healing process by monitoring the diffusion of Eu^3+^ and Tb^3+^ (Figure 2b). They pointed out that a mixed interphase of more than 100 nm and less than 175 nm was required to achieve complete recovery of the mechanical properties. This study provides a direct proof for the self-healing process that molecular-level diffusion is essential to heal the cracks. Furthermore, it establishes a framework for further investigation of the healing process in intrinsic self-healing system based on the dynamic bonds.

## 3. Recent Advances of Self-Healing Elastomer with Fast Healing Speed

As the self-healing polymers developed rapidly, the self-healing elastomers with a fast healing speed (<2 h), even real-time (<30 s), are attracting intensive interest because of its substantial applications in flexible electronics for a long-term practical use with high stability and safety. According to the healing process of the self-healing polymers above mentioned, the healing rate is mainly related with the wetting and molecular-level diffusion stages in the form of the exchange rate of dynamic bonds and the diffusion rate of polymer chains at the interfaces [66]. The factors such as the application of mechanical force on the healing interfaces, the increment of healing temperature and external stimuli (e.g., UV light irradiation, visible light, and microwave), are important for the fast self-healing speed. Furthermore, the reversible dynamic bonds with a fast exchange rate, such as hydrogen bonds, have been also widely studied to fabricate a self-healing elastomer with fast healing speed.

### 3.1. Thermally Triggered Fast Self-Healing Elastomers

It is well documented that the migration of polymer chains is increasing with the increase of temperature as well as the self-healing capability [70]. For example, Feng et al. [71] developed a fast self-healing elastomer based on hydrogen bond by emulsion interfacial polymerization. The obtained elastomer can recover its initial tensile strength after 96 h healing at room temperature, while it takes only 2 h at body temperature. Thus, it is an effective method to improve the healing efficiency and speed through increasing the healing temperature, especially for a fast self-healing elastomer with high mechanical performance [72,73].

**Figure 3:**
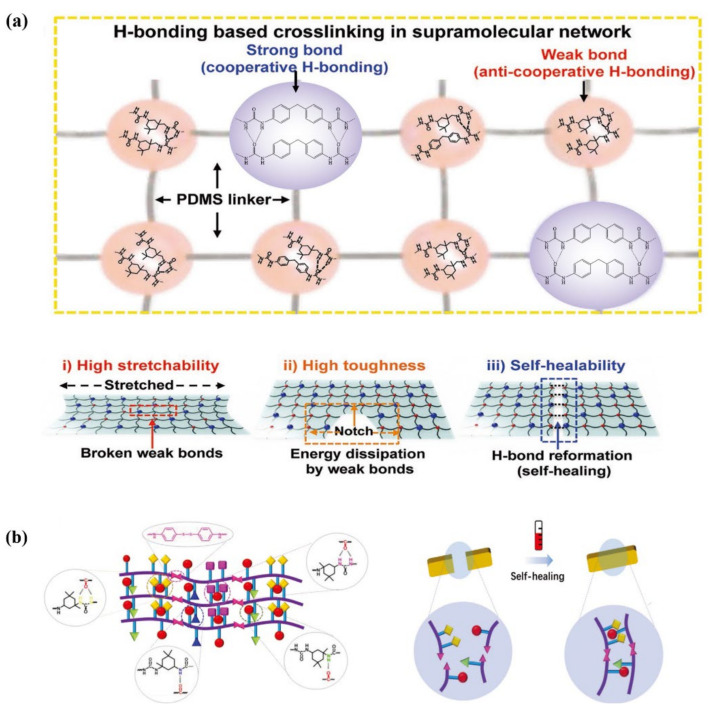
(**a**) Possible hydrogen bonding combinations for strong bond and weak bond, respectively, and schematics of a stretched polymer film (i), notched film (ii), and healed film (iii). (**b**) Schematic diagrams of ideal elastomer structure and self-healing mechanism. Reproduced with permission [74,75]. Copyright 2018 WILEY-VCH Verlag GmbH & Co. KGaA. Weinheim. Copyright 2021 Wiley-VCH.

Based on the self-sorting chemistry, Bao et al. [74] reported a new elastomer design to construct unprecedented supramolecular network composed of varying composition of well-defined strong and weak hydrogen bonds linked by the hydrophobic PDMS backbone. Anti-cooperative weak hydrogen bonds afforded the elastomer with high stretchability and toughness (see Figure 3a). The abundant dynamic hydrogen bonds and the low *T_g_* of PDMS backbone endow the elastomer with a fast self-healing capability with ca. 86% recovery of its tensile strength after 1.5 h healing at 60 °C, and even with healing ability in artificial sweat and under water. Along with its easy processability, a capacitive strain-sensing e-skin with high toughness and robustness against damage is conceptualized and successfully fabricated. This facile molecular design provides a framework for self-healing elastomers with high mechanical performance, which is expected to be applicable in various polymer structures.

Aromatic disulfide bonds possess rapid reaction kinetics at mild temperature, which promotes the migration of polymer chains, and are widely investigated in the fabrication of fast self-healing elastomers with high tensile strength. Zhang et al. [75] has fabricated mechanically robust self-healing polyurethane with disordered structure through one-step synthesis procedure. The steric hindrance of asymmetric alicyclic structure endows the polymer chains or chain segments with sufficient mobility and retaining of mechanical properties simultaneously, while the soft segments maintain the toughness and supply high-density hydrogen bonds to improve tensile strength simultaneously (see Figure 3b). All in one, a unique dual dynamic cross-linked network with high-density hydrogen bonds is formed and the homogeneous structure without micro-phase separation achieves the outstanding mechanical properties (tensile strength of 41 MPa and toughness of 104 MJ m^−3^) and excellent self-healing ability simultaneously at mild temperature (more than 80% recovery of its initial tensile strength of completely cut sample for just 1 h healing at 60 °C). In addition, Table 2 summarizes the research of self-healing polymers triggered by thermal based on different dynamic interactions, where t is the healing time and T represents the temperature at which the self-healing process is conducted.

### 3.2. Light Triggered Fast Self-Healing Elastomers

As thermally triggered self-healing system can heal the microcracks with the recovery of most initial properties at elevated temperatures [83], light irradiation is another effective method to realize self-healing capability [84]. Compared with the heat stimulus, the utilization of light to trigger the healing process exhibits following advantages: (i) accurately exert a stimulus on the desired position or/and area, avoiding the influence of it on the properties of other parts, (ii) make the inaccessible damage heal available through remote operation of the healing process, and (iii) tailor the light wavelength to selectively match the specific reaction conditions for the practical requirements. These features largely expand the applications of self-healing materials in the fields where the heating stimulus is unavailable [85].

Graphene possesses excellent electrical and thermal conductivity as well as good microwave and infrared (IR) absorbing capacity [86]. Thus, incorporation of graphene into appropriate polymeric matrices can not only improve the tensile strength but also realize the fast self-healing. Huang et al. [87] fabricated a fast self-healing elastomer (which only needed several minutes to achieve 99% healing efficiency) with multi-channels healing ability through simply integrating few-layer graphene (FG) with thermoplastic polyurethane (TPU). The incorporation of FG gives the composite excellent IR absorption. Moreover, the outstanding conductivity of FG transfers the Joule energy into TPU matrix effectively. Consequently, in the self-healing process, FG acts as a nano-heater and transfer unit to generate the required energy and then transport the energy to the matrix efficiently. Thus, the FG-TPU samples can be heated rapidly and homogeneously under IR light, resulting in a fast diffusion and re-entanglement of the TPU chains at the broken interface of a damaged composite for quickly healing of the fractures. Similar to FG, carbon nanotube also exhibits such properties. Yang et al. [88] has reported a fast self-healing vitrimer composite composed of carbon nanotube and epoxy, which can heal after only 30 s irradiation.

UV light is widely utilized to facilitate the self-healing speed of the elastomers through light-to-heat conversion as well. For example, Yan et al. [89] utilized 2-ureido-4-pyrimidone (UPy) as dynamic and light-to-heat conversion moieties to fabricate UV triggered fast self-healing elastomer that exhibits a high healing efficiency of 86% after 1 min irradiation. The elastomer can be heated up to 63 °C. On the other hand, UV light can also accelerate the healing speed by facilitating the exchange reaction of dynamic covalent bonds. Zhao and co-workers [90] have investigated the influence of disulfide group content on the self-healing speed of UV triggered healing film. They found that the self-healing speed increased with the increment of disulfide group. The tensile strength of the damaged sample reached up to 15.9 MPa after UV irradiation for 40 s as the sample temperature increased to 46 °C. The fact that the damaged sample cannot be healed by solely heating up to 46 °C for 90 s indicates that the UV irradiation indeed facilitates the exchange reaction of disulfide bonds.

Compared with UV light, visible light is a relatively milder and can never cause unnecessary damage to polymer matrix. Visible light is merely suitable to trigger the reversible covalent bonds with low bond energy, such as thiuram disulfide (240 kJ mol^−1^) and diselenide (172 kJ mol^−1^) bonds, which are most widely chosen to construct self-healing elastomers with longer healing times (>4 h) [90,91]. The ditelluride bond with lower energy (126 kJ mol^−1^) compared with disulfide and diselenide bonds is suitable for the design of visible-light triggered self-healing elastomers with fast healing speed [92]. Fan and co-workers [93] have fabricated a fast self-healing elastomer triggered by visible-light with high healing efficiency based on dynamic ditelluride bonds. Quadruple hydrogen-bonded UPy moieties are additionally introduced to improve the mechanical performance of it. The resultant elastomer exhibits excellent mechanical properties (tensile strength of ca. 20 MPa and toughness of 105 MJ m^−3^). The damaged sample can recover 85.6% of the original strength after 10 min irradiation under visible light with a slight increase of temperature to 40 °C. The recovery of only 21% original strength of the reconnected sample in the dark for 10 min reveals that the visible light is a primary factor for the healing of the dynamic ditelluride bond.

### 3.3. Mechanical Force Enhanced Fast Self-Healing Glassy Polymers

**Figure 4:**
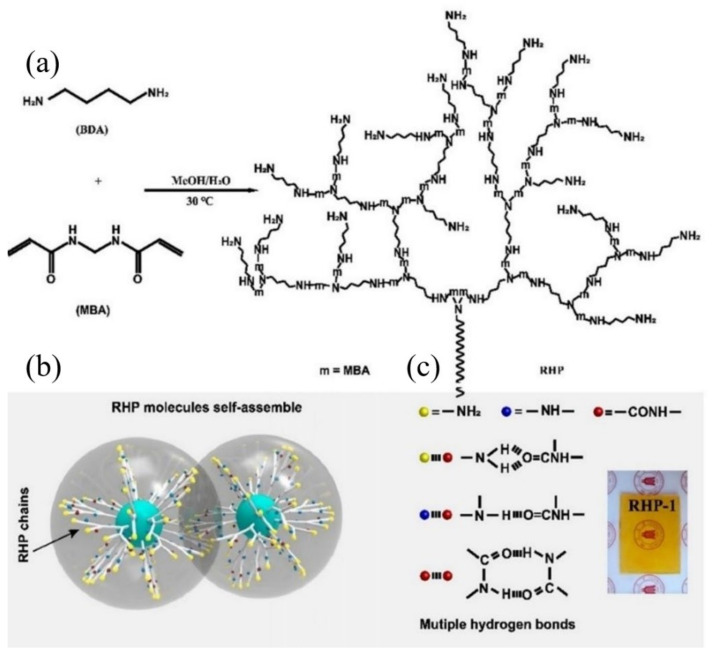
Design concept and synthesis of RHP. (**a**) Chemical route to the synthesis of RHP through Michael addition between MBA and BDA at 30 °C. (**b**) Schematic diagram of interactions between RHP molecules. (**c**) Multiple hydrogen bonds of RHP molecules. (Inset) A photograph of RHP-1. Reproduced with permission from [96]. Copyright 2020 National Academy of Sciences.

Glassy polymers are widely used in automobiles, airplanes, aerospace vehicles, etc., as structural materials, due to their high mechanical properties, e.g., strength, modulus, and stiffness. However, the high mechanical performance is highly dependent on the frozen cross-linked network within the polymer, and, also, makes them hard to heal the mechanical crack autonomously because of the restricted migration of polymer chains [84,94,95]. Consequently, nine-tenths of glassy polymers with self-healing ability reported so far need the assistance of external energy, e.g., light, heat, or solvent, and a long time to achieve healing. Recently, Wang and co-workers [96] reported a room temperature self-healing hyperbranched polymer (RHP), which displayed a fast self-healing speed with the mechanical strength recovered up to 5.5 MPa after only 1 min healing. Unlike conventional linear polymers, RHP possesses a 3-D spatial configuration composed of internal backbone structures and external branching units with different end groups. Moreover, the internal parts are closely packed, and thus display poor mobility, while the branched external units exhibit a relatively high degree of mobility. As mentioned above, if self-healing moieties are elaborately introduced on the external branched units and end groups, the obtained RHP may possess self-healing capability. Based on this concept, a series of RHPs through one-pot Michael addition have been fabricated as shown in Figure 4. All the *T_g_s* of the fabricated RHPs are well above room temperature, indicating that the obtained RHPs are all in the glassy state at ambient temperature. According to the dielectric loss spectra, all RHPs display three relaxation processes, termed as β-relaxation, γ-relaxation, and δ-relaxation, respectively. Among them, δ-relaxation exhibits the shortest relaxation time, followed by γ-relaxation and β-relaxation. In particular, all three processes can occur below *T_g_*. This implies that the branched chain units and end groups of RHPs are mobile in the glassy state, which is responsible for the self-healing capability at room temperature.

**Figure 5:**
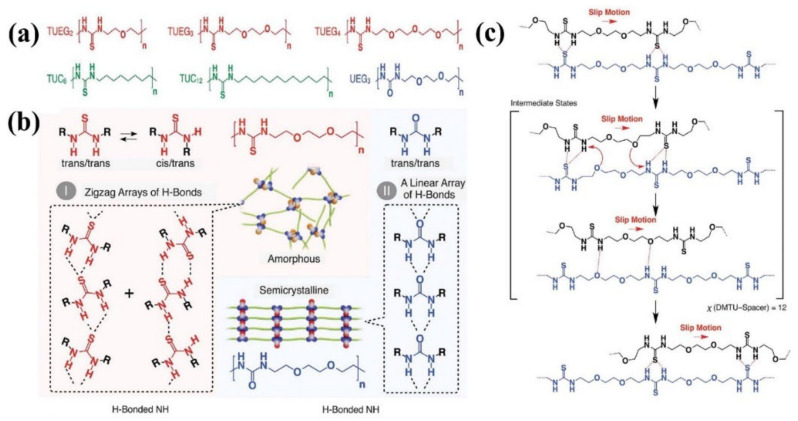
(**a**) Schematic structures of poly(ether-thioureas) with diethylene glycol (TUEG2), triethylene glycol (TUEG3), and tetraethylene glycol (TUEG4) as spacers; schematic structures of poly(alkylene-thioureas) with octamethylene (TUC8) and dodecamethylene (TUC12) chains as spacers; and schematic structure of a poly(ether-urea) with triethylene glycol (UEG3) as a spacer. (**b**) Schematic representations of the H-bonding modes of thiourea and urea. (**c**) Proposed mechanism of how the exchange of H-bonded thiourea pairs in TUEG3 is enhanced. Reproduced with permission from [97]. Copyright (2018) American Association for the Advancement of Science.

As aforementioned, wetting can impact the subsequent self-healing, and even determine the kinetics of healing process. Researchers usually apply a certain pressure on the damaged surfaces to obtain good wetting during the healing process, in which the compressive force on the healing interfaces may attenuate the activation energy barrier of dynamic bond exchange that promotes healing in a manner of accelerating the dynamic exchanges. The facilitating self-healing process in glassy self-healing polymer has also been discovered. It is well known that polymer chains with high molecular weight are always heavily entangled and hardly diffuse. Aida et al. [97] have reported a dense hydrogen bond cross-linked polymer with low molecular weight that exhibits high tensile strength and rapid self-healing ability. A series of poly(ether-thiourea) glassy polymers with low molecular weight have been synthesized by one-pot polycondensation as shown in Figure 5a. As illustrated in Figure 5b, the densely hydrogen-bonded thiourea arrays within the polymers are geometrically nonlinear (zigzag hydrogen-bonded array), i.e., less ordered, and thus exhibit the amorphous feature. Benefitting from the less ordered dense hydrogen bonds, TUEG3 exhibits the highest tensile strength of 35 MPa and can recover its original tensile strength completely with the assistance of a constant pressure of 1 MPa at 28 °C for 1 h. On the basis of the data acquired from the rheological technique, the relaxation time of TUEG3 ranges from 107 s (on the order of months) to 105 s (on the order of days) in the temperature range of 24 °C to 32 °C, much longer than the healing time. This means that the fast self-healing behavior of TUEG3 is not a consequence of the migration of polymer chains rather than the much rapid exchange of the hydrogen-bonded thiourea pairs, which results in the interpenetration of polymer chains at the damaged interface upon constant pressure. Furthermore, slipping motion of polymer chains occurs through exchange of hydrogen-bonded pairs, which is suggested to be facilitated by the ether oxygen atoms serving as temporal hydrogen bond acceptors as presented in Figure 5c. At last, the essential structural elements for the construction of robust self-healing polymer with fast self-healing ability are proposed as follows: (i) relatively short polymer chains that permit greater segmental motions, (ii) tight cross-links by a large number of hydrogen bonds for better mechanical properties, (iii) less ordered nonlinear hydrogen-bond arrays that hinder the crystallization, and (iv) implemented mechanism to facilitate the exchange of hydrogen-bonded pairs.

**Figure 6:**
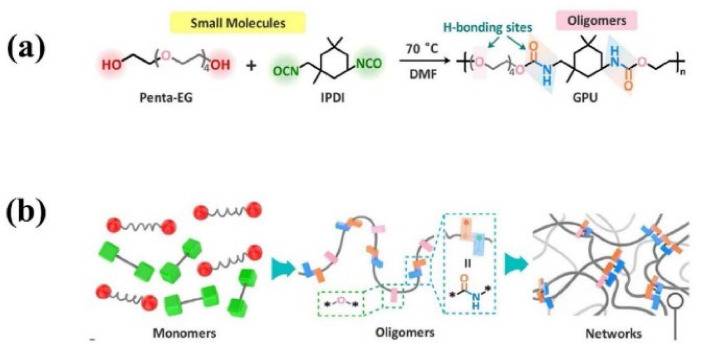
(**a**) Molecular structure, synthetic process, and illustration of GPU polymer and (**b**) cross-linked network inside of it. Reproduced with permission from [98]. Copyright 2021 Wiley-VCH GmbH.

Similar to the principle illustrated above, Fu and co-workers [98] have developed a highly colorless and transparent glassy polyurethane (GPU) with low molecular weight by one-pot polycondensation reaction of penta-ethylene glycol (Penta-EG) and isophorone diisocyanate (IPDI) (Figure 6). The dense hydrogen bonds within GPU are responsible for the unprecedentedly robust stiffness with a high Young’s modulus of 1.56 GPa. Importantly, the asymmetric alicyclic structure of IPDI supplied a high degree of steric hindrance resulting in the loose packing of the adjacent urethane moieties, which endows the assembled hydrogen bonds with high mobility after dissociation even below *T_g_*, and accelerates the re-formation of the broken networks. Thus, the yielded GPU displays a fast self-healing capability despite of its rigid nature. Although the realization of fast self-healing of GPU is also assisted by a constant pressure of 1 MPa, the healing mechanism of it is not totally the same as TEUG3 reported by Aida et al. [97] The fast self-healing ability of GUP is also not dominated by the dynamic diffusion of polymer chains. Actually, the freshly cut surface of GPU can firmly stick to other substrates, such as glass and modified aluminum under gentle pressure, while the non-freshly-cut surfaces cannot under the same condition. The investigation of the molecular configurations at the surface, interior, and freshly cut interfaces of GPU via X-ray photoelectron spectroscopy (XPS) indicates that the percentage of free hydrogen-bond is about 17.25% at the surface, while the value increases to 36% within the interior layer, and to 78.64% in the freshly cut interfaces. Thus, the plenty dissociated urethane and ether moieties in freshly cut interfaces are responsible for the high adhesion performance, which re-bond with their complementary moieties rapidly under the constant pressure to ensure the fast self-healing of the material. Consequently, the high degree of steric hindrance of isophorone urea moieties endow the urethane moieties with high mobility below *T_g_*, which benefit the large amount of cleaved hydrogen bond moieties in the damaged interfaces to reform with their counterparts and thus restore the mechanical properties quickly at room temperature. For this reason, the damaged GPU only needs 10 min at room temperature upon constant pressure of 1.0 MPa to achieve a mechanical strength of 7.74 MPa (50% of its initial strength), but the healing trails cannot disappear after 10 min healing because of the interfacial molecular-level interpenetration is restricted below *T_g_*.

### 3.4. Room-Temperature Fast Self-Healing Elastomers

**Figure 7:**
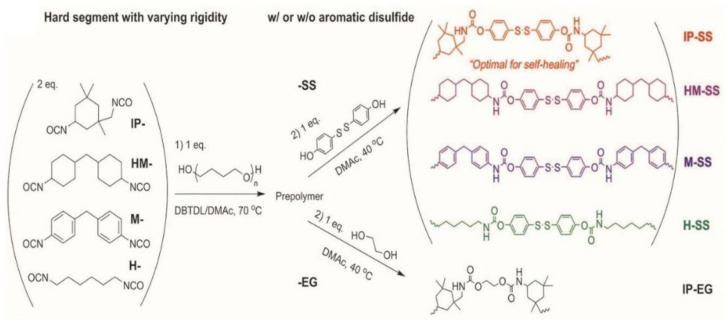
Synthetic routes to TPUs with four different diisocyanates (i.e., IP, HM, M, and H) and two chain extenders (i.e., SS and EG). The TPUs are designated as X–Y, where X and Y denote the abbreviation of the diisocyanate monomer and chain extender, respectively. Reproduced with permission from [104]. Copyright 2017 WILEY-VCH Verlag GmbH & Co. KGaA, Weinheim.

Compared with the self-healing elastomers illustrated above, elastomers that can spontaneously self-heal at room temperature without external stimuli are highly desired, due to the facile condition of self-healing process [99]. Dynamic noncovalent bonds or dynamic covalent bonds with low activation energy, e.g., aromatic disulfide, and sufficient mobility of polymer chains are essential factors for the design of room temperature self-healing elastomers. However, these factors always yield elastomers with insufficient mechanical performance [100]. Thus, it is challenging to design room temperature self-healing elastomers with high mechanical properties. To this end, ingenious stratagems have been proposed, such as multiphase design [101], binary filler strategy [102], and cartilage-inspired nanostructure [34] to achieve both high self-healing efficiency and excellent mechanical strength at room temperature. On the other hand, healing time is another important factor for the self-healing elastomers, especially in practical applications, the shorter the better. A great many works were devoted to design self-healing elastomer with fast self-healing speed [103]. Park and co-workers [104] have fabricated an elastomer that can achieve a healing strength of ca. 6 MPa with a healing efficiency of more than 75% after 2 h healing. To disclose the relationship between structure and properties, a series of TPUs, termed IP-SS, HM-SS, M-SS, H-SS, and IP-EG, with different linking parts have been synthesized (shown in Figure 7). The asymmetric alicyclic structure of IPDI improves the mobility of polymer chains, resulting in a higher healing efficiency of IP-SS and IP-EG than others. The TPU cannot heal at room temperature in the absence of SS indicates the key role of aromatic dynamic disulfide in the room temperature self-healing process.

**Figure 8:**
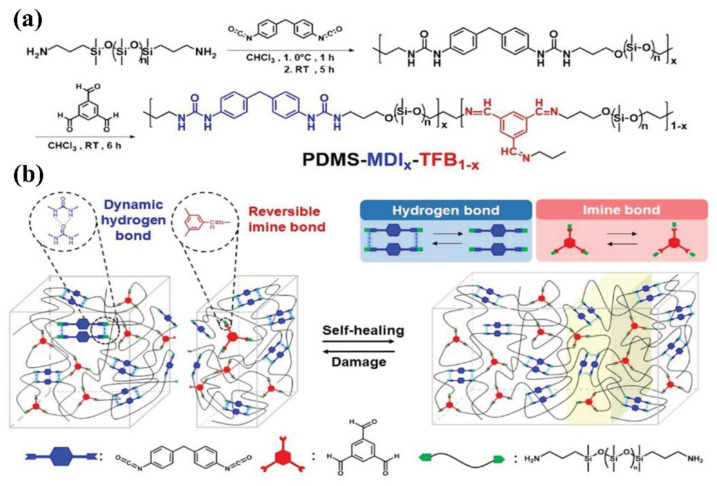
(**a**) Synthetic route to prepare PDMS-MDIx-TFB1-x self-healing elastomer. (**b**) Schematic illustration of ideal structure of PDMS-MDI_x_-TFB_1−x_ based on the synergistic effect of reversible weaker imine bonds and stronger hydrogen bonds. Reproduced with permission from [93]. Copyright 2021 Wiley-VCH GmbH.

Utilization of the synergistic effect of different dynamic bonds is another effective way to construct fast self-healing elastomers, such as combined non-covalent system and non-covalent/covalent system [105,106,107]. Plus, the combined non-covalent/covalent system may produce novel functional properties. For example, Kuo and co-workers [108] reported a novel fast self-healing elastomer based on the synergistic effect of Schiff-based imine bond and hydrogen bond, which can heal not only at ambient condition but also under water. The synthetic process and the dynamic interaction inside it are shown in Figure 8. The synergistic effect of the hybrid dynamic cross-linked network combined with high mobility of PDMS chains endows the elastomer with fast self-healing ability and satisfactory tensile strength upon suffering damage. Specifically, the elastomer can recover its original mechanical properties after 1 h healing under water. It is speculated that the double entropic penalty caused by the decrement of conformational entropy of PDMS and unfavorable changing of van der Waals energetic interaction induced by the reversible reaction between water molecule and Schiff-based imine are responsible for the fast self-healing capability of the obtained elastomer.

**Figure 9:**
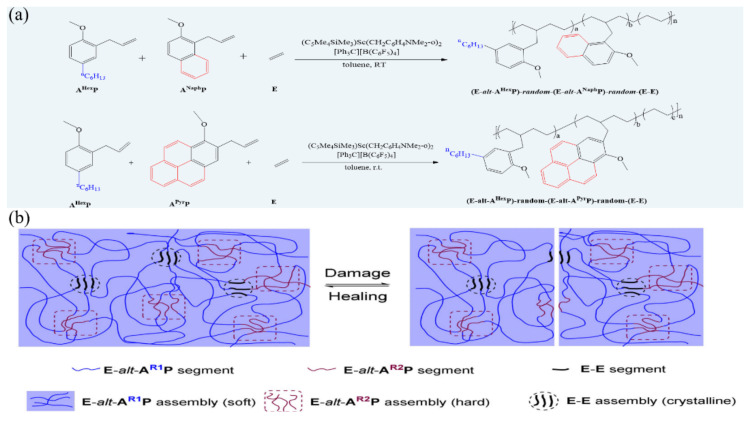
(**a**) Terpolymerization of ethylene (E), hexylanisyl propylene (AHexP), and methoxynaphthyl propylene (ANaphP)/methoxypyrenyl propylene (APyrP) by a half-sandwich scandium catalyst. (**b**) Representation of multiphase morphology of a E-AR1P-AR2P terpolymer and its self-healing mechanism. E = ethylene, A^R1^P = hexylanisyl propylene (AHexP), A^R2^P = methoxynaphthyl propylene (ANaphP), or methoxypyrenyl propylene (APyrP). Reproduced with permission from [112]. Copyright (2021) Wiley-VCH GmbH.

Van der Waals interactions have been known for more than two centuries, while the existence of van der Waals interactions in polymeric materials is verified decades ago [109]. The research works focusing on self-healing polymers based on van der Waals interactions are, however, rarely [110,111]. In a recent work, Hou and co-workers [112] have fabricated a fast self-healing elastomer based on van der Waals interaction by terpolymerization of a non-polar olefin and two different polar functional olefins in a controlled fashion as shown in Figure 9a, which exhibits fast (within 1 h) self-healing ability and high mechanical properties at room temperature because of the formation of a nanoscale 3-D network structure through multi-phase separation of nanodomains of the crystalline E-E segments and the hard amorphous (E-alt-ANaphP or E-alt-APyrP) segments from the flexible E-alt-AHexP segments matrix via van der Waals interactions (Figure 9b). As suffering damage, the re-aggregation of the E-E segments and E-alt-ANaphP or E-alt-APyrP segments will result in the re-association of the network structure and consequently repair of the damage. The exceptionally fast self-healing may be ascribed to the very high mobility of the flexible E-alt-AHexP segments, which can induce an unusually rapid re-assembly of the E-E and E-alt-ANaphP or E-alt-APyrP segments.

In recent years, a new kind of self-healing elastomer was developed and attracted increasing interest because of the ultra-fast self-healing ability which only need few minutes, even few seconds to completely heal [113,114,115]. The framework about ultra-fast healing elastomers with low mechanical strength (less than 1 MPa) has first been established by Bao et al. [116]. They reported a composite material comprised of supramolecular polymers with embedded nickel nanostructured microparticles which can completely restore the mechanical strength after 10 min healing, but only a ca. 40% recovery in strain. The key factors for this ingenious design are: (i) the sufficient hydrogen bonds network in supramolecular polymer to ensure the dissociation of hydrogen bonds prior to covalent bond broken, and (ii) low *T_g_* facilitates the rearrangement, wetting, and diffusion of polymer chains on the damaged interfaces. Similar to this design philosophy, Zhang and co-workers [17,18,19] have developed three kinds of fast self-healing nanocomposite elastomers based on natural rubber, cellulose nanocrystals, and GO-nanosheet, which can almost completely recover their mechanical properties after 2 min, 15 s, and 10 s healing at room temperature without external stimuli. Three different dynamic non-covalent bonds are used, i.e., metal-ligand coordination bond, multiple hydrogen bond, and electrostatic interaction, respectively. Moreover, Wu and co-workers [117] have designed a homogeneous ultrafast self-healing elastomer composed of PDMS backbone with high mobility and plenty of hydrogen bonds formed between boric acid (BA) and dithiothreitol (DTT), which can completely recover its mechanical properties after 30 s healing at ambient environment but with a tensile stress of only 0.4 MPa. The tensile strains of the elastomer are unchanged as the strain rate increased from 50 mm min^−1^ to 500 mm min^−1^, indicating that the elastomer possess high mobility of polymer chains and fast exchange rate of hydrogen bonds that are responsible for the ultrafast self-healing ability. Unlike the work reported by Bao et al. [116], these works exhibit excellent self-healing ability in tensile strain as well as tensile strength, while the underlying mechanism of this difference is still unknown.

**Figure 10:**
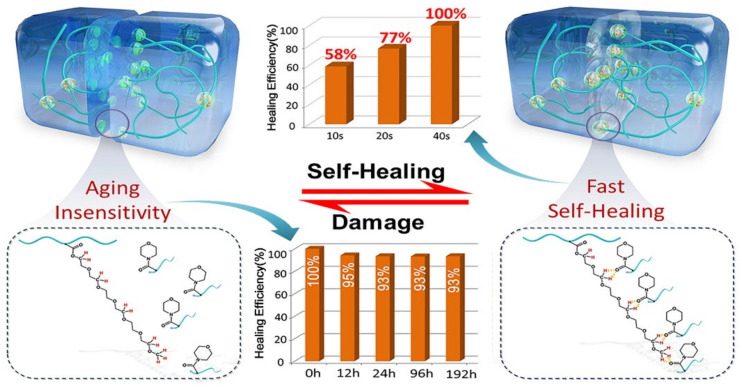
The self-healing properties and the healing mechanism of our fast self-healing elastomers Reproduced with permission from [121]. Copyright 2020 American Chemical Society.

**Figure 11:**
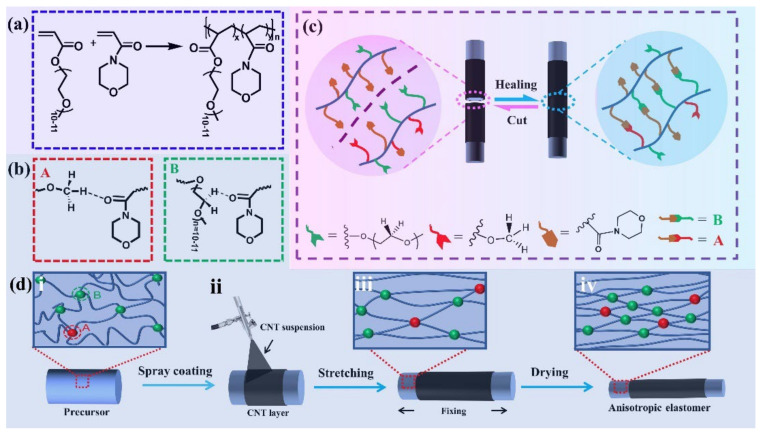
Illustrating the preparation and self-healing process of ultrafast self-healing elastomers coated with CNT layer and the corresponding self-healing mechanism. Free radical polymerization of acryloylmorpholine (ACMO) and PEG 480 methyl ether acrylate (**a**) to get cross-linked precursor hydrogel (i in (**d**)), which can be dried under stretching (iii in (**d**)) to produce self-healing elastomer (iv in (**d**)) with anisotropic hierarchical structure and the reversible interaction of it were shown in A and B of (**b**). CNT coated self-healing electrodes was obtained by directly spray coating of CNT suspension on the surface of precursor hydrogel (ii), and then suferring fixed drying process as shown in (iii and iv). (**c**) The self-healing process of the obtained elastomer. Reproduced with permission from [122]. Copyright 2022 Elsevier B.V.

Apart from the ultrafast self-healing elastomers with tensile strengths of lower than 1 MPa, fast self-healing elastomers with high tensile strength (>1 MPa) have also been reported. For example, Choi and co-workers [118] have reported a colorless, robust bio-based supramolecular polymer through one pot polycondensation reaction which only need 60 s to recover its initial mechanical properties with a tensile strength of 2.8 MPa. Moreover, the Young’s modulus of the elastomer is up to 340 MPa, which is the same as low-density polyethylene. On the other hand, we have also devoted to designing the fast self-healing elastomers with high mechanical strength [119,120,121,122]. In a previous work, a fast self-healing elastomer has been fabricated through the free radical polymerization using poly (ethylene glycol) 400 methyl ether acrylate (mPEG400-acrylate) and acryloylmorpholine (ACMO) as the raw materials, which exhibits a fast self-healing ability and high mechanical strength (only need 40 s to achieve complete healing with a tensile strength of 4.2 MPa as shown in Figure 10). According to the healing theory, the polymer chains on the damaged interface can rearrange, and the chains with low molecular weight species are preferred to migrate to the surface during the rearrangement process. For this reason, the short PEG chain (the side chain of the obtained polymer) will migrate to the interfaces upon damage due to the high mobility of PEG chains and accelerate the re-association of the broken hydrogen bonds. This results in a fast self-healing speed and has been clarified by studying on the reformation kinetics of hydrogen bonds within the elastomer. Recently, the side chains of the elastomer have been increased slightly by using mPEG480-acrylate as materials instead of mPEG400-acrylate, which not only provides more bonding sites but also improves the mobility of side chains. As expected, the thus obtained elastomer needs only 5 s to achieve a tensile strength of ca. 4 MPa with a healing efficiency of 90%. An elastomer with anisotropic structures has been further fabricated by simply drying the precursor hydrogel under fixed strain (Figure 11). Moreover, the resulting anisotropic elastomers exhibit enhanced mechanical strength of 8.4 MPa, which can restore 86% of its original mechanical properties after 10 s healing at ambient temperature without external stimuli. CNT layer with hierarchical structure can also be easily prepared by directly spraying the CNT suspension onto the surface of precursor hydrogel before drying under strain, which can be used as a sensor to detect human motions from the subtle to large scale.

## 4. Conclusions and Outlook

This minireview focuses on the construction of fast self-healing polymers through physical and chemical approaches. Based on exploring and understanding the fundamental mechanism underlying the healing process of self-healing polymers, many stimuli, such as thermal, irradiation, and pressure, were utilized to facilitate the healing speed of the polymers. Compared to fast self-healing polymer triggered by stimulus, the autonomic fast self-healing polymers are unique placed to use in flexible electronics. The last couple years, therefore, have brought significant advances in the fabrication of autonomic fast self-healing elastomers. Ingenious stratagems have developed to endow polymer chains with both fast migration rate and abundant reversible dynamic interactions which can facilitate the self-healing process, in order to realize fast self-healing ability. Fast, even real-time, self-healing elastomers with high mechanical strength at ambient condition without the need of external stimuli have thus been reported. Despite brilliant advances, there are still some obstacles restricting practical applications of the self-healing elastomers: (i) It is essential to bring the damaged interfaces together immediately after being damaged because the healing efficiency is attenuated with the increment of aging time. (ii) For completely fractured samples, certain pressure must be applied on the damaged surface prior to healing.

According to the healing process proposed by Wool et al. [43,56], chain segments with low surface tension and molecular weight preferentially migrate to the interfaces in the surface rearrangement stage. It may be a new research direction for design of self-healing elastomers with new functions through controlling molecular weight, molecular weight distribution, and functional-group placement. On the other hand, it is well known that the complete healing cannot be achieved until the finish of diffusional interpenetration of polymer chains in the damaged interfaces. However, the real-time self-healing elastomers can restore its original mechanical properties in a few seconds, and it seems that the polymer chains hardly migrate in such short time. Consequently, it is necessary to disclose the underlying mechanism of fast self-healing, which still remains a challenge in this field. In addition, the evaluation of healing efficiency of self-healing polymers is mainly dependent on characterization of mechanical properties, such as tensile strength, tensile strain, toughness, and Young’s modulus before cut and after healing. The investigation of healing process in micro or nanoscale, even in molecular level events is highly desired.

## Figures and Tables

**Table 1:** External pressure assistant self-healing polymers based on dynamic covalent bonds.

Dynamic Bonding	Pressure	Temperature (°C)	Time (h)	Ref.
Disulfide bond	10 bar	70	7	[59]
Disulfide bond	30 kPa	70–90	1	[60]
Disulfide bond	1 bar	70	7	[61]
Disulfide bond	0.01 MPa	120	5	[62]
Disulfide bond	1 bar	70	7	[63]
Boron-based bond	Slight force	40	6	[64]

**Table 2:** Thermally triggered self-healing polymers based on different dynamic interactions.

Matrix	Healing Mechanism	T (°C)	t	Ref.
poxy/TPU	Hydrogen bond	80	10 min	[76]
PLA/PEG/HPDMS	Diels-Alder reaction	120	100 s	[77]
TPUs	Disulfide bond	50	1 h	[73]
Polyacrylate composite	Hydrogen/Disulfide bonds	60	5 min	[78]
PCL-PU	Shape memory effects	65	2 h	[79]
PU	Diels-Alder reaction	130	2 h	[80]
TPU	Disulfide bond	70	60 s	[81]
Polyacrylate composite	Coordination bond	60-80	10 min	[20]
PCL	Disulfide bond	60	1 h	[82]

## Data Availability

Not applicable.
